# Comparative regional morphometric changes in human uterine artery before and during pregnancy

**Published:** 2012-10-13

**Authors:** Moses M Obimbo, Julius A Ogeng'o, Hassan Saidi

**Affiliations:** 1 Department of Human Anatomy, University of Nairobi, Kenya

**Keywords:** Morphometry, pregnancy, reproductive, uterine artery

## Abstract

**Introduction:**

Uterine artery undergoes structural modifications at different physiologic states. It is expected that due to its unique course, hemodynamic stresses in the vessel would vary resulting in differences in arterial dimensions. The objective of this study was to investigate regional morphometric changes in the human uterine artery.

**Methods:**

Twenty four uterine arteries (12 each from non-gravid uteri and gravid uteri) were obtained during autopsy after ethical approval from women aged between 21 to 47 years. Sections from proximal, middle and distal segments of the artery taken within 72 hours were processed for paraffin embedding, sectioned and stained with Mason's Trichrome. Micrographs of the slides were analyzed using Scion Image Multiscan software. Data were entered into and analyzed with Statistical Programme for Social Sciences.

**Results:**

The pregnancy related increase in diameter and wall thickness are most pronounced in the proximal segment. In the distal segment, however, wall thickness reduces significantly (p < 0.05). Intimal thickness was lesser in pregnancy compared to non-gravid state in all the segments.

**Conclusion:**

Regional morphometric changes in the uterine artery during pregnancy may be designed to regulate blood flow to the uterus and placenta during pregnancy.

## Introduction

The uterine artery, during pregnancy undergoes structural adaptations [1, 2] which involve changes in wall structure and luminal size [3]. These changes are associated with a threefold increase in blood supply to the uterus [4, 5] and are important for determining fetal outcome [6, 7]. They appear to be triggered by hemodynamic stresses to which the artery is subjected [8] and their extent varies with its orientation [9]. What is not clear is how the uterine artery dimensions change along the artery given its unique course. This study investigated regional morphometry of uterine artery in pregnancy and used uterine artery from non gravid uteri for comparison.

## Methods

**Figure 1:**
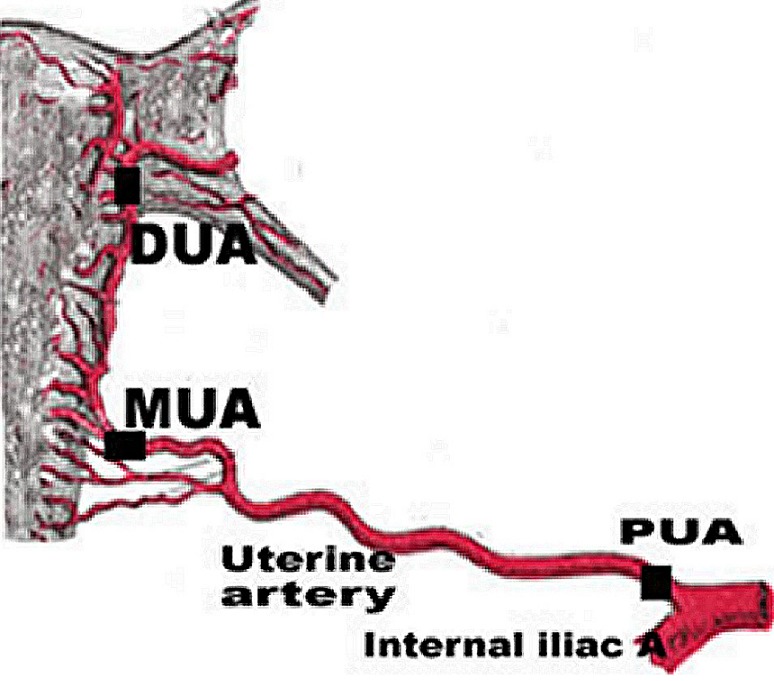
Harvesting sites from uterine artery (PUA: Proximal Uterine artery, MUA: Middle Uterine artery, DUA: Distal Uterine artery)

**Figure 2:**
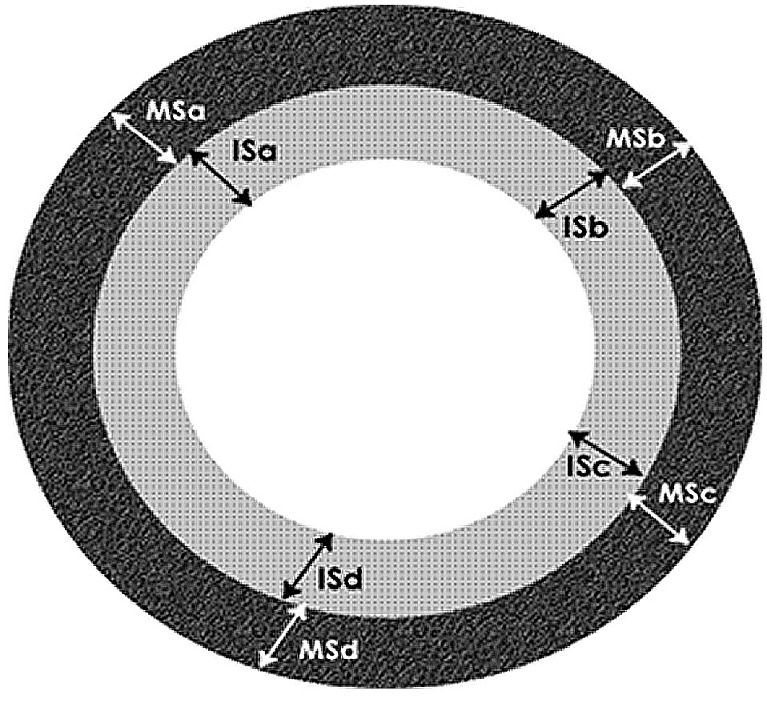
Morphometric measurements of uterine artery

**Figure 3:**
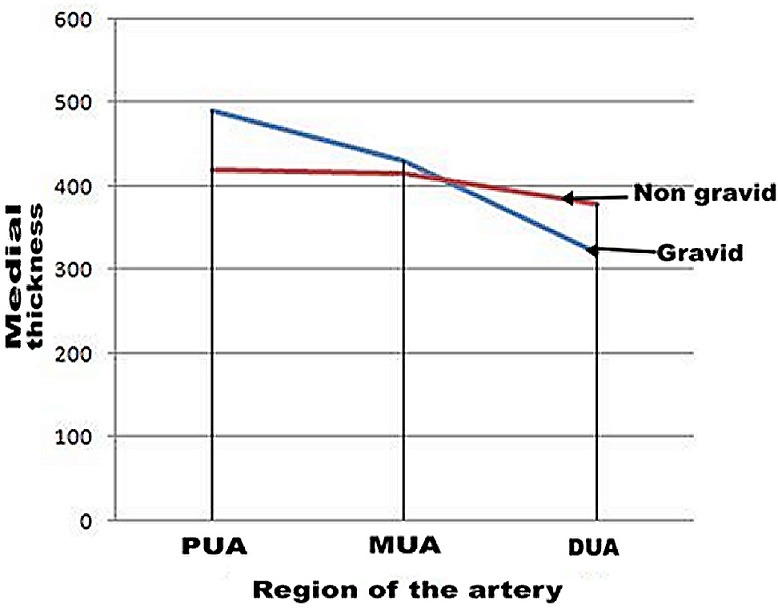
Medial thickness in different regions of uterine artery by gravid status (PUA: Proximal Uterine artery, MUA: Middle Uterine artery, DUA: Distal Uterine artery)

Twenty four uterine arteries (12 from non-gravid uteri of women in reproductive period and 12 from gravid uteri) obtained within four hours of death from women and mothers who died mainly from road traffic accidents and complications during child birth. The women were aged between 21 and 47 years with no history of chronic ailments. The arteries were obtained from postmortem material in Chiromo, Nairobi City and Kenyatta National Hospital mortuaries. The uterine arteries from non-gravid uteri were of women who had no history of previous births. Conversely, those from gravid uteri were of women in whom the current pregnancy was either the first or second. In the later group, two samples were from uteri in 2nd and the rest were in the 3rd trimester of pregnancy. Approval to use the material was granted by Kenyatta National Hospital Ethics and Research Committee. Informed Consent was obtained from the relatives of the deceased. Extended pelvic abdominal incision was made into the pelvis and peritoneum opened to expose the common iliac trunk. It was followed to the internal iliac and its anterior and posterior branches identified. The uterine artery was identified and five millimeter sections taken from three regions: Proximal (PUA) immediately after it was given off from the anterior branch of internal iliac artery; Middle (MUA) at the cervix before it curved to ascend on the uterus and distal (DUA) at the junction between the uterus and the uterine tubes (Figure 1,Figure 2,Figure 3).

The segments were routinely processed for paraffin embedding and sectioning [10]. Seven micrometer thick sections cut using a Leitz Wetzlar sledge microtome (SM2400, Germany) were stained with Masson's Trichrome. A total of 72 blocks were prepared, 24 from each segment (PUA, MUA and DUA). Every second block from each group (gravid and non gravid) was selected to a total of 6 blocks. The blocks were then sectioned. A total of one hundred and twenty eight slides from each block were made. Every fourth slide to a total of thirty two slides from each block was selected through purposive sampling. One hundred and ninety two slides from each region for both gravid and non gravid uteri were then studied for morphometry.

They were examined under Leica Light microscope (BME model, Germany) at a constant magnification of x40. Photomicrographs were taken using Fujifilm Finepix A900 (9.0 Megapixels) digital camera and images analyzed using Scion Image Multiscan software. Luminal diameter, intimal and medial thickness was obtained as follows; the region around the lumen was traced using Multiscan and the collapsed artery was transformed into a dilated physiological state that is circular. The length obtained was taken as the circumference of the circle and the diameter for the lumen computed using the mathematical formula shown: D= C/pi. Where D= diameter, C = circumference and pi = 3.142. The media was defined as the area between the internal elastic lamina and the external elastic lamina. Four random points were selected and the average thickness was computed by dividing the sum of the four thickness sizes with by [11]. The extent of the intima was defined as between the lumen and the internal elastic lamina. Four random points were selected and the average length computed.

The wall thickness: luminal ratio was calculated by dividing the wall thickness with the diameter of the artery at a specified region. Data were analyzed using Statistical Programme for Social Sciences (SPSS) 11.5 and presented in form of digital macrographs and tables.

## Results

All of the 24 uterine arteries were studied. The mean luminal diameter of the uterine artery increased significantly in all the segments, but was most marked in the proximal (22%) and least in the middle (14.5%) segments (Table 1).

**Table 1:** Mean luminal diameters proximal, middle and distal of uterine arteries segments by gravid status (N = 192)

Segment of uterine artery	Gravid status	Mean luminal diameter (mm) ± SD	Average % increase in diameter
Proximal	Gravid	1.94 ± 0.27	22
	Non gravid	1.59 ± 0.13	
Middle	Gravid	1.74 ± 0.76	14.5
	Non gravid	1.52 ± 0.12	
Distal	Gravid	1.42 ± 0.33	20.3
	Non gravid	1.18 ± 0.23	

Medial thickness increased markedly in the proximal (16.7%) than in the middle (3.4%) segments. In the distal segment, however, there is a significant reduction in the medial thickness compared to a non gravid state (Figure 1).

The decline in the medial thickness in pregnancy compared to nonpregnant state in the in the distal segment was statistically significant (p= 0.039). Intimal thickness showed a proximo-distal decline both in pregnancy and in non gravid state. The average thickness during pregnancy was, however, smaller (107.5 µm + 2.9µm) compared to a non gravid uterine artery (157.5µm + 7.5µm). The differences observed in intimal thickness between the two groups was not statistically significant (p= 0.234) (Table 2). The wall thickness: luminal diameter ratio remained uniform along the length of the artery but was lower in pregnancy (Table 3).

**Table 2:** Mean intimal thickness of the uterine artery segments by gravid status

Segment of uterine artery	Gravid status	Mean intimal thickness (µm ±SD)
Proximal	Gravid	127 ± 12
	Non gravid	188 ± 9
Middle	Gravid	101 ± 14
	Non gravid	151 ± 16
Distal	Gravid	82 ± 7
	Non gravid	138 ± 9

**Table 3:** Wall thickness: luminal diameter ratios of segments of uterine artery by gravid status

Segment of uterine artery	Gravid status	Ratio
Proximal	Gravid	3:10
	Non gravid	4:10
Middle	Gravid	3:10
	Non gravid	4:10
Distal	Gravid	3:10
	Non gravid	4:10

## Discussion

Observations of the present study reveal that as reported in the literature [3, 12] the diameter of the uterine artery increases during pregnancy. This has been described as an adaptive response to increased blood flow within the uterine artery [13, 14], which results from enhanced vasodilator response, decreased vasoconstrictor response, alterations in active and passive mechanical properties of the uterine artery wall [15]. A higher percentage increase in the lumen of the distal segment in comparison to the middle segment may imply that this zone creates a low pressure zone and hence allows for pooling of blood [16]. The middle segment has the least increase in the diameter probably because of it giving off a cervical branch which makes the artery generally narrower at this point. Indeed, when arteries branch, there is an overall decrease in their luminal diameter [17]. The overall widened lumen allows accommodation of a threefold increased blood flow [18] to meet the demand of the developing conceptus [19]. Failure of the uterine artery to adapt in this way during pregnancy usually leads to a poor pregnancy outcome [3, 20]. This study, however, did not take into account the age at pregnancy and parity states of the women, factors that could have had an effect on the histomorphology of the uterine artery.

Increased uterine wall thickness is most marked in the proximal segment. This increase is related to both hyperplasia and hypertrophy of smooth muscles [1, 21]. Functionally, medial hypertrophy may act as a regulator of blood supply to a target organ [22]. Accordingly, the proximal segment of the uterine artery may be the “regulator zone” which adjusts blood flow to the uterus. Secondly, the media both in the proximal and the middle segment may provide a valve like mechanism to the upstream flow of blood and thus prevent backflow.

In the distal segment, the tunica media of the uterine artery of the gravid uterus was significantly thinner than that of the corresponding segment in the non-gravid uteri. This medial thinning of the distal segment, hitherto, unreported may be an adaptation to allow efficient blood flow with minimal impedance to the decidual arteries that supply the placenta [16]. Indeed, previous reports on decidual arteries have demonstrated replacement of smooth muscles within the media with amorphous material [23]. This structural modification of the media of the arteries supplying the placenta is thought to facilitate the expansion of these vessels to provide increased blood flow as pregnancy advances and their occlusion and collapse after parturition [24]. The functional consequence of this modification may be to ensure an adequate and efficient flow of maternal blood to the placenta, thus enhancing the survival of the fetus.

Intimal thickness showed a proximo-distal decline. The proximal segment of the uterine artery had the largest evidence of thickening. This implies that this segment experiences the most wall shear stress, wall pressure, or particle deposition as it bears the highest pressure effect and turbulence after receiving its blood from a major vessel. This pattern has been observed in regions where arteries branch from the main trunk and are useful indicators of future atherosclerotic disease [25]. The middle segment followed in thickness probably due to the branching off of the cervicovaginal artery. This being a smaller branch, the observation of reduced intimal thickening is expected. The distal segment had the least intimal thickening suggesting that low blood pressure and subsequent low wall shear stress at this region did not favor deposition of particles to result in intimal thickening. This concept has been explored before [26].

The thickness of the intima in pregnancy was reduced compared to the non gravid period. Similar observations were made by Kamiya (1989) [27], who demonstrated difficulties in identifying gravid sclerosis characterized by a thickening of the intima and a lamination of the internal elastic lamina in pregnancy and early postpartum period. Similar changes have also been described in uterine arteries of gravid guinea pigs and sow [28, 29]. These cyclical changes appear to be under the influence of hemodynamics, growth factors and hormones [27]. Changes in the hormonal profile and an increase in the volume of blood flowing to the uterus could explain the decreased thickness of the intima. High levels of oestrogen during pregnancy with action in the uterine artery could be responsible for the adaptation seen [30]. Indeed, some arteries are able to respond to changes in the internal milieu over a very short period of time [31]. Intimal thickening in reproductive period outside pregnancy could be explained because of the lower levels of oestrogen as compared to pregnancy. Crawford et al., 1997 [32] showed significant portions of intimal hyperplasia in women in reproductive age group and worse in post menopause when the protective effect of oestrogen was at the lowest.

The wall thickness to luminal ratio in all the segments remained constant during pregnancy. The ratio was; however, lower during pregnancy compared to non-gravid state because of the sharper increase in luminal diameter. When true values were considered, there was a progressive decline in the wall thickness parameters as opposed to the lumen in pregnancy. In non gravid uteri wall thickness parameters progressively showed constant decline as opposed to the rapid decline of the luminal diameter dimensions giving it a higher ratio. Previous literature has not described the changes in the wall thickness to luminal diameter ratio in different segments of the uterine artery but the general concept is in tandem with what has been described before about the morphology of arteries [2, 3]. The resultant effect of the increased ratio during pregnancy is to increase overall blood flow to the uteroplacental bed and reduce uterine vascular resistance to flow.

## Conclusion

Regional morphometric changes in the uterine artery during pregnancy may be designed to regulate blood flow to the uterus. The higher medial thickness in the proximal region may confer a regulator role, while the reduced wall thickness with a wider lumen in the distal segment functions to pool blood and permit low pressure regulated placental perfusion.
